# Impact of Acute Metal Stress in *Saccharomyces cerevisiae*

**DOI:** 10.1371/journal.pone.0083330

**Published:** 2014-01-09

**Authors:** Dagmar Hosiner, Susanne Gerber, Hella Lichtenberg-Fraté, Walter Glaser, Christoph Schüller, Edda Klipp

**Affiliations:** 1 Department of Applied Genetics and Cell Biology, UFT-Campus Tulln, University of Natural Resources and Life Sciences (BOKU), Vienna, Austria; 2 AG Computational Time Series Analysis, Institute of Computational Science, Faculty of Informatics, Università della Svizzera italiana, Lugano, Switzerland; 3 Theoretical Biophysics Humboldt-Universität zu Berlin, Berlin, Germany; 4 AG Molekulare Bioenergetik, IZMB, Rheinische-Friedrich-Wilhelms-Universität, Bonn, Germany; 5 Institute of Medical Biochemistry, MFPL, Medical University of Vienna, Vienna, Austria; German Cancer Research Center, Germany

## Abstract

Although considered as essential cofactors for a variety of enzymatic reactions and for important structural and functional roles in cell metabolism, metals at high concentrations are potent toxic pollutants and pose complex biochemical problems for cells. We report results of single dose acute toxicity testing in the model organism *S. cerevisiae*. The effects of moderate toxic concentrations of 10 different human health relevant metals, Ag^+^, Al^3+^, As^3+^, Cd^2+^, Co^2+^, Hg^2+^, Mn^2+^, Ni^2+^, V^3+^, and Zn^2+^, following short-term exposure were analyzed by transcription profiling to provide the identification of early-on target genes or pathways. In contrast to common acute toxicity tests where defined endpoints are monitored we focused on the entire genomic response. We provide evidence that the induction of central elements of the oxidative stress response by the majority of investigated metals is the basic detoxification process against short-term metal exposure. General detoxification mechanisms also comprised the induction of genes coding for chaperones and those for chelation of metal ions via siderophores and amino acids. Hierarchical clustering, transcription factor analyses, and gene ontology data further revealed activation of genes involved in metal-specific protein catabolism along with repression of growth-related processes such as protein synthesis. Metal ion group specific differences in the expression responses with shared transcriptional regulators for both, up-regulation and repression were also observed. Additionally, some processes unique for individual metals were evident as well. In view of current concerns regarding environmental pollution our results may support ongoing attempts to develop methods to monitor potentially hazardous areas or liquids and to establish standardized tests using suitable eukaryotic a model organism.

## Introduction

Metals and metalloids are an integral part of our environment and widespread in nature. Organisms become exposed to metals either through natural sources or, more recently, through anthropogenic sources such as the use of metals and metal compounds as fungicides and disinfectants. Some metals are intrinsically toxic like cadmium, arsenic, and mercury. Others such as copper, manganese, or zinc are required in trace amounts for essential cellular functions but become toxic in excess quantities [1] (Table S1 in File S1).

Since metals cannot be degraded or modified like toxic organic compounds they persist in cells and interfere with cellular homeostatic pathways [2]. Metal toxicity is the consequence of several effects on cellular and organismal level including oxidative stress [3], alteration of enzyme and protein function [4], [5], [6], [7], lipid peroxidation, and DNA damage [8], [9], [10], [11]. To preserve the delicate balance between essential and toxic levels of certain metal ions such as copper and iron, cells utilize sophisticated mechanisms to regulate uptake, sequestration to sub-cellular compartments and complexes, as well as detoxification [12], [13].

*S. cerevisiae* senses and responds to a variety of environmental conditions like nutrient depletion, temperature, osmotic- and oxidative stress, and a number of chemically diverse toxicants such as hydrogen peroxide, the superoxide-generating drug menadione, the sulfhydryl-oxidizing agent diamide, or the disulfide-reducing agent dithiothreitol [14], [15], [16]. Most toxic metal ions are not directly detected because no specific cellular sensors exist. Thus, exposed cells respond to the secondary induced damage. In yeast cells and other microorganisms stress responses are affected by rapid adjustment of gene expression patterns. This capacity is amendable to analysis via expression profiling and positions yeast, with the restriction of being a simple eukaryotic cell, as a suitable system to measure cellular responses to metal toxicity and to deduce detoxification strategies.

A substantial amount of information concerning the exposure of yeast cells to toxic metals and metalloids is available [17]. However, these data result largely (with the exception of Jin et al., [16]) from specific studies and are difficult to compare. Jin and coworkers [16] conducted the first comparative metal stress response study that focused on sustained metal stress in yeast cells. In contrast, we launched a comparative study of acute metal stress. We investigated the immediate effects of metal ions with significant relevance to human health in *S. cerevisiae* (Table S1 in File S1). The experimental rationale concentrated on short-term exposure to moderate metal ion concentrations and the examination of transcriptional profiles obtained after 30 minutes exposure to Ag^+^, Al^3+^, As^3+^, Cd^2+^, Co^2+^, Hg^2+^, Mn^2+^, Ni^2+^, V^+^, and Zn^2+^. Our study revealed a different but characteristic pattern compared to Jin et al. [16], suggesting a more transient type of responses. The most common immediate defense responses against acute metal stress were metal-specific oxidative stress response and protein degradation. Analyses of the regulatory network architecture revealed for certain groups of metal ions shared response patterns such as activation of specific detoxification processes or repression of ribosomal biogenesis.

## Materials and Methods

### Metal Toxicity Assays

For determination of the Lowest Observable Effect Level (LOEL) and the half maximal Effective Concentration (EC50) upon metal treatment in *S. cerevisiae*, the effect of 10 biologically relevant metal ions (Ag^+^, Al^3+^, As^3+^, Cd^2+^, Co^2+^, Hg^2+^, Mn^2+^, Ni^2+^, V^+^, and Zn^2+^) on yeast growth was examined. BY4741 (MATa *leu2 ura3 his3 met15 can1*) overnight cultures were diluted in liquid YPD (2% yeast-extract, 1% peptone, 2% glucose, pH 6.4) to an optical density OD_600_ of 0.15 and grown to 0.8 at 30°C. The cultures were then supplemented with increasing concentrations of dissolved metal salts (AgNO_3_, AlCl_3_, AsCl_3_, CdCl_2_, CoCl_2_, HgCl_2_, MnCl_2_, NiSO_4_, VCl_3_, and ZnCl_2_) and incubated for 12 hours at 30°C. Yeast growth was monitored via optical density (OD_600_, Hitachi U2000) at 2 h intervals. From individually obtained graphical growth curves EC50 values and LOEL, defined as the concentration inducing 5% growth inhibition were optically determined. Each metal toxicity analysis consisted of one control and up to 5 test concentrations. Expression profiling (EP) was performed with equipotent ( = equal in effect, between 50 and 300 differentially expressed genes in 30 min) metal concentrations in the range between LOEL and EC50. For each metal ion at least three independent replicate tests were carried out and standard deviations were up to a negligible range of about ±3%. Results are listed in table 1.

**Table 1:** Metal toxicity assays in *S. cerevisiae*.

Metal	LOEL	EC50	EP
**AgNO_3_**	20 µM	100 µM	40 µM
**AlCl_3_**	400 µM	3 mM	500 µM
**AsCl_3_**	50 µM	500 µM	200 µM
**CdCl_2_**	1 µM	10 µM	2 µM
**CoCl_2_**	100 µM	400 µM	100 µM
**HgCl_2_**	20 µM	75 µM	30 µM
**MnCl_2_**	1 mM	1.75 mM	1.5 mM
**NiSO_4_**	1 mM	2 mM	1.5 mM
**VCl_3_**	200 µM	4 mM	500 µM
**ZnCl_2_**	1 mM	2.5 mM	1.5 mM

(LOEL) Lowest Observable Effect Level and (EC50) half maximal Effective Concentration, *de facto* concentration for EP (expression profiling).

### Phenotypic Screening

Over-night cultures of wild-type BY4741 and the disruptant BY4741 Δ*stb5* (EUROSCARF) were diluted in liquid YPD and grown from OD_600_ 0.15 to 0.8 at 30°C. The exponentially growing yeast cells were then spotted in 10-fold dilutions onto solid YPD containing increasing concentrations of dissolved metal salts (AgNO_3_, AsCl_3_, CdCl_2_, CoCl_2_, HgCl_2_, MnCl_2_, NiSO_4_, VCl_3_, and ZnCl_2_), as well as on YPD control plates lacking metal ions. All plates were incubated for 48 hours at 30°C, and the yeast spots were visually scored to determine growth restriction.

### Microarrays

Microarrays were conducted according to MIAME [18] standards. Over-night cultures were diluted with 50 ml fresh liquid YPD medium to an OD_600_ of 0.15 as start inoculum, grown to OD_600_ 0.8 and treated with the indicated concentrations of dissolved metal salts (EP in table 1), incubated for 30 min at 30°C, washed and frozen. Total RNA was prepared by the hot acidic phenol extraction method with three chloroform extractions. Microarrays (obtained from Microarray Centre Toronto, Ontario, CAN) containing PCR fragments of 6144 predicted *S. cerevisiae* open-reading-frames (ORFs) spotted in duplicates were used for expression profiling essentially as recommended by the manufacturer. Fluorescently labeled cDNA was synthesized from 15 µg of total RNA by oligo dT-primed polymerization using 200 U Superscript II (Invitrogen, Carlsbad, CA) and Cy3-CTP or Cy5-CTP (GE Healthcare, Waukesha, WI). Labeled cDNAs were pooled and purified by the Cy Scribe GFX purification kit (GE Healthcare). Hybridization was performed for 14–18 h in DigEasyHyb solution (Roche Diagnostics, Basel, Switzerland) with 0,1 mg/ml salmon sperm DNA (Sigma, St Louis, MO) at 37°C. Microarrays were washed three times in 1×SSC, and 0.1% SDS at 50°C for 10 min followed by 1 min in 1×SSC and 0.1%×SSC at room temperature and spun to dryness. All microarray experiments were performed in triplicate.

### Microarray Analysis

The microarrays were scanned using an Axon GenePix 4000B scanner (Molecular Devices, Sunnyvale, CA). The image data were quantified using the GenePix Pro4.1 software (Molecular Devices). Raw data are available under the ArrayExpress (http://www.ebi.ac.uk/arrayexpress/) accession E-MEXP-2985 and E-MEXP-2987. The data preprocessing and the differential expression tests were performed with Bioconductor, particular the Limma package (Package limma version 3.8.3) [19]. For the present evaluations the background correction method “normexp” [20] was applied. With this method a convolution of normal and exponential distributions is fitted to the foreground intensities by using the ambient signal as a covariate. The expected signal given by the observed foreground becomes the corrected intensity. An offset-value of 50 was chosen as is recommended [20], [21]. This approach resulted in a smooth monotonic transformation of the already background corrected intensities. Typical problems such as negative corrected intensities and high variability of low intensity log-ratios were thus avoided [20], [21].

All spots with missing values as well as “bad”-spots with illegal shape were zero weight allocated for all further normalization and analysis steps. The corrected intensities were used to form the log-ratio and the average log-intensity for each spot. Normalization was performed using the robust spline normalization [22] which is an empirical Bayes compromise between Print-tip and Global Loess normalization with 5-parameter regression splines used in place of the Loess curves.

Afterwards a quantile normalization [23] between all arrays ensured that the average intensities show the same empirical distribution across arrays and across channels. The quantile normalization approach first ranked data on each array and substituted data of the same rank across all arrays by the mean of the data.

Differentially expressed genes were identified as follows: For each gene six data points were available arising from the three performed experimental replicates (independent arrays) where each of the replicates contained two spots with the same probe. A linear model was fitted to the expression of each probe [19], [24]. Replications of the same treatment were merged in the calculation due to the consideration of dye-swaps. Duplicated spots within a single array were evaluated using a pooled correlation method to make full use of the information [24]. Genes were ranked in order of differential expression of the stressed samples in comparison to the reference sample in each experiment. All genes that appeared significantly differentially expressed after the normalization process (showing at least a +/−50% deviation from normal conditions) were taken into account for further analysis. The identification of commonly expressed genes was performed using the R script “overlapper.r” (R version 2.13.2; http://faculty.ucr.edu/~tgirke/Documents/R_BioCond/My_RScripts/over Lapper.R).

Cluster analyses [25], [26] were performed using cluster3 and visualized with TreeView [27]. For further data evaluation clustering algorithms using the Matlab Bioinformatics toolbox (Matlab: R2012) were applied to identify groups of genes with similar expression profiles for the time point “30 minutes” after metal exposure. Significant associations to gene ontology (GO)-terms and regulatory associations were obtained by GO Term Finder provided by SGD (http://www.yeastgenome.org/cgibin/GO/goTermFinder.pl) and T Profiler (http://www.t-profiler.org/).

## Results and Discussion

### Metal toxicity analyses in liquid culture

The presented experiments focused on the early-on transcriptional changes in response to single dose acute toxicity testing by metal exposure to avoid adaptation. To define concentration thresholds for metal ions toxicity in *S. cerevisiae* cells under our culture conditions, the LOEL and the EC50 of the selected metals were determined (Table 1). Exponentially growing cultures supplemented with increasing concentrations of dissolved metal salts were incubated for 12 hours and yeast growth was measured via optical density every 2 hours. In the following all metals mentioned refer to the respective metal ions in aqueous solution. According to the presumed toxicity and functional/physiological role of the metals the LOEL and EC50s varied substantially. Of the applied 10 metal ions Cd^2+^ was found the most toxic with a LOEL of 1 µM and an EC50 of 10 µM. Al^3+^ exhibited with 400 µM the highest LOEL and V^+^ with 4 mM the highest EC50.

For expression profiling (EP; Table 1) LOEL and EC50 were taken as lower and upper threshold. Equipotent exposure concentrations were taken to induce transcriptional changes of between approximately 50 and 300 genes within 30 min. Such moderate stress conditions as well as the brief exposure for 30 min enabled the detection of early-on responses without entering the general (environmental) stress response (ESR) comprising a multitude of expression responses [15].

### Expression profiles under conditions of moderate, acute metal stress

**Figure 1:**
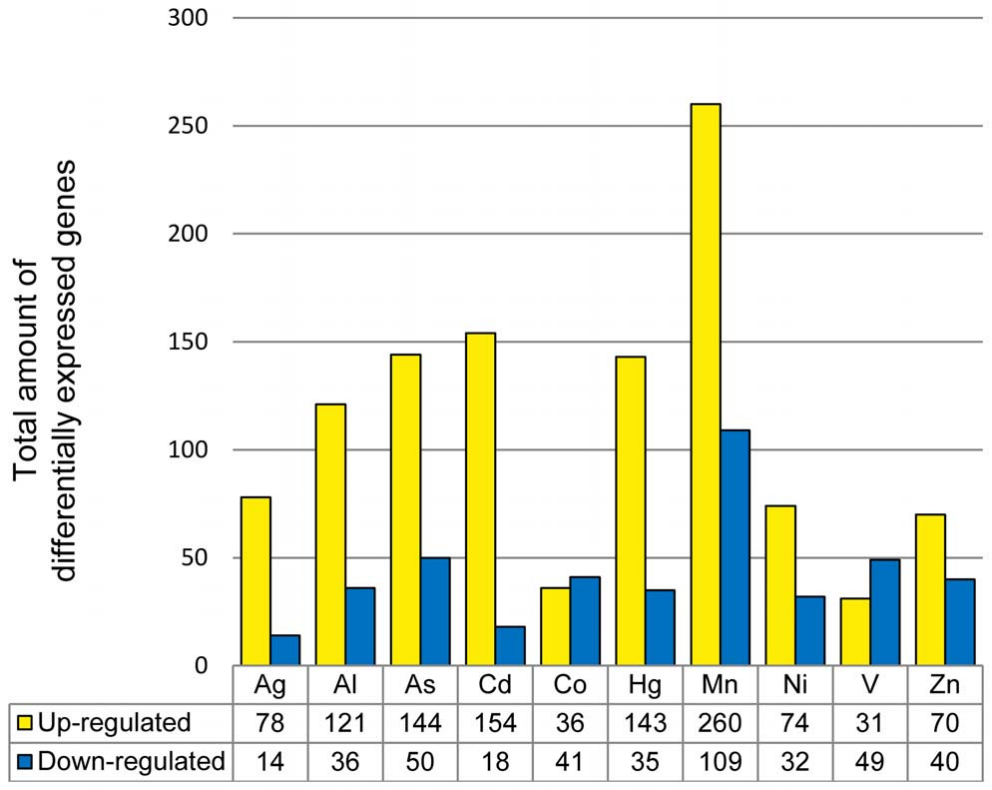
Total number of differentially expressed genes. Descriptive summary of the total number of genes differentially expressed by a factor greater 50% (yellow) or minor 50% (blue) upon treatment with indicated concentrations of metal ions compared to the untreated control; total numbers of up- and down-regulated genes upon the distinct metal stress conditions are indicated in the figure. In total 740 genes were up-regulated, and 283 genes were down-regulated. Detailed information is provided in TS 1, 2, and 3 in File S2.

Microarrays were analyzed as described. A minimum of 50% deviation from the untreated control was defined as significant change of expression. According to this criterion, 740 genes were up-regulated and 283 genes were down-regulated (Figure 1; additional information is provided in Tab Sheet (TS) 1, 2, and 3 in File S2) and selected for further analyses. Mn^2+^ treatment induced the maximal response with 260 up- and 109 down-regulated genes, followed by Cd^2+^ with 154 up- and 18 down-regulated genes, whereas Co^2+^ exhibited the least response with 36 up- and 41 down-regulated genes. Ag^+^ treatment resulted in the up-regulation of 78 genes, comparable to Ni^2+^ and Zn^2+^. Of the three metals with no biological role (Table S1 in File S1) Ag^+^ and Cd^2+^ exposure induced the lowest number of down-regulated genes, namely 14 and 18. The third, Hg^2+^, induced down-regulation of approximately twice the number (35 genes) and, near Cd^2+^ up-regulation of 143 genes. Overall transcriptional responses to the individual metal treatments were within a range of approximately 50 to 300 differentially expressed genes and further subjected to comparisons of the metal stress response patterns.

### Hierarchical clustering of the expression profiles

Under the assumption that co-regulated genes respond to the same regulatory control mechanisms or are regulated by similar signals [28], [29] overlaps between the transcriptional profiles upon different metal ion exposures were investigated via hierarchical clustering to identify similarities in the expression patterns.

Via pair-wise comparison of all experiments the number of shared or overlapping genes (Table 2; additional information is provided in TS 4 and 5 in File S2) was determined. Next, genes that were expressed differentially solely under one metal stress condition were identified (Table 3; additional information is provided in TS 6 in File S2). Again, the metals Ag^+^, Cd^2+^ and Hg^2+^ induced the lowest number of uniquely down-regulated genes, namely 0, 3, and 1, respectively. The repressed genes are involved in ribosomal subunit biogenesis, transcription initiation and purine accumulation.

**Table 2:** Pair-wise comparison of the 10 metal stress conditions.

	Ag^+^	Al^3+^	As^3+^	Cd^2+^	Co^2+^	Hg^2+^	Mn^2+^	Ni^2+^	V^3+^	Zn^2+^
**Ag^+^**	78/−14	1	28	32	1	34	42	29	3	6
**Al^3+^**	−3	121/−36	7	4	1	7	1	2	3	5
**As^3+^**	−7	−1	144/−50	53	3	35	28	27	2	8
**Cd^2+^**	−5	−1	−9	154/−18	7	56	35	23	1	6
**Co^2+^**	−2	−1	−1	−1	36/−41	1	12	8	1	9
**Hg^2+^**	−8	−1	−17	−11	−1	143/−36	50	24	2	5
**Mn^2+^**	−7	−5	−17	−8	−4	−20	260/−109	21	0	10
**Ni^2+^**	−5	−1	−10	−5	−7	−9	−5	74/−32	1	10
**V^3+^**	−2	−16	−4	−3	−3	−4	−5	−3	31/−49	7
**Zn^2+^**	−1	−8	−1	−1	−5	−1	−4	−2	−12	70/−40

Matrix of the number of shared (overlapping) genes after pair-wise comparison of all differentially expressed genes upon 10 metal expositions. The diagonal line illustrates the total amount of genes significantly differentially expressed in response to the particular metal ions (up/down-regulated). Detailed information is given in TS 4 and 5 in File S2.

**Table 3:** Uniquely expressed genes under different metal stress conditions.

	Ag^+^	Al^3+^	As^3+^	Cd^2+^	Co^2+^	Hg^2+^	Mn^2+^	Ni^2+^	V^3+^	Zn^2+^
**Up-reg.**	12	81	44	34	17	30	145	17	14	32
**% induced**	15%	66%	30%	22%	47%	21%	56%	23%	45%	46%
**Down- reg.**	0	12	21	3	25	1	68	10	14	21
**% repressed**	0%	33%	42%	17%	61%	3%	62%	31%	29%	52%

Numbers of up- or down-regulated genes under the distinct metal ions; percentage referred to the total numbers of differentially ex- or repressed genes provided in table 3. Detailed information is given in TS 6 in File S2.

Upon query of the most frequently induced genes in our dataset it was found that no single gene was induced under all 10 metal stress conditions (Table 4; additional information is provided in TS 7 in File S2). 17 up-regulated and 4 down-regulated genes out of in total 1023 differentially expressed genes were changed under at least five treatments. *TRX2*, encoding an oxidoreductase of the thioredoxin system that protects cells against oxidative stress was found as the most frequently up-regulated gene (Ag^+^, As^3+^, Cd^2+^, Hg^2+^, Mn^2+^, Ni^2+^, and Zn^2+^). *NSR1*, coding for a protein involved in ribosomal biogenesis was the most frequently down-regulated gene (Ag^+^, As^3+^, Cd^2+^, Hg^2+^, Mn^2+^, and Ni^2+^). Though hierarchical clustering revealed no specific early-on metal detoxification pathway for all tested metals, however gene expression patterns showed some common processes for distinct groups of metal ions.

**Table 4:** Genes expressed or repressed in at least five metal stress conditions.

Counts	SID	GS	Conditions	Up/Down
7	YGR209C	TRX2	Ag^+^-As^3+^-Cd^2+^-Hg^2+^-Mn^2+^-Ni^2+^-Zn^2+^	Up
6	YMR251W-A	HOR7	Ag^+^-As^3+^-Hg^2+^-Mn^2+^-Ni^2+^-Zn^2+^	Up
6	YML100W	TSL1	Ag^+^-As^3+^-Cd^2+^-Hg^2+^-Mn^2+^-Ni^2+^	Up
6	YLR303W	MET17	Ag^+^-As^3+^-Cd^2+^-Hg^2+^-Mn^2+^-Ni^2+^	Up
6	YKL103C	LAP4	Ag^+^-As^3+^-Cd^2+^-Hg^2+^-Mn^2+^-Ni^2+^	Up
6	YEL060C	PRB1	Ag^+^-As^3+^-Cd^2+^-Hg^2+^-Mn^2+^-Ni^2+^	Up
6	YDL124W	NA	Ag^+^-As^3+^-Cd^2+^-Hg^2+^-Mn^2+^-Ni^2+^	Up
6	YGR159C	NSR1	Ag^+^-As^3+^-Cd^2+^-Hg^2+^-Mn^2+^-Ni^2+^	Down
5	YHL040C	ARN1	Cd^2+^-Co^2+^-Mn^2+^-Ni^2+^-Zn^2+^	Up
5	YPL154C	PEP4	Ag^+^-As^3+^-Hg^2+^-Mn^2+^-Ni^2+^	Up
5	YNL160W	YGP1	Ag^+^-As^3+^-Cd^2+^-Hg^2+^-Ni^2+^	Up
5	YMR173W	DDR48	Ag^+^-As^3+^-Cd^2+^-Hg^2+^-Mn^2+^	Up
5	YHR071W	PCL5	Ag^+^-Cd^2+^-Hg^2+^-Mn^2+^-Ni^2+^	Up
5	YDL022W	GPD1	Ag^+^-As^3+^-Cd^2+^-Mn^2+^-Ni^2+^	Up
5	YCL040W	GLK1	Ag^+^-As^3+^-Cd^2+^-Hg^2+^-Mn^2+^	Up
5	YNL208W	NA	Ag^+^-Cd^2+^-Hg^2+^-Mn^2+^-Ni^2+^	Up
5	YNL134C	NA	Ag^+^-As^3+^-Cd^2+^-Hg^2+^-Mn^2+^	Up
5	YCL042W	NA	Ag^+^-As^3+^-Cd^2+^-Hg^2+^-Ni^2+^	Up
5	YPR124W	CTR1	As^3+^-Cd^2+^-Hg^2+^-Ni^2+^-V^3+^	Down
5	YML056C	IMD4	As^3+^-Cd^2+^-Hg^2+^-Mn^2+^-Ni^2+^	Down
5	YLR175W	CBF5	Ag^+^-As^3+^-Cd^2+^-Hg^2+^-Mn^2+^	Down

Genes significantly up- or down-regulated (Up/Down) in at least 5 of the 10 experimental metal stress conditions; “Counts” number of conditions under which the respective gene was differentially expressed; SID (systematic identification), GS (gene symbol); “Conditions” declare the combinations of experimental conditions under which the respective gene showed response. Detailed information is given in TS 7 in File S2.

### Transcription factors under acute metal stress

To identify potential regulators affected by acute metal stress the statistical prediction tool T-Profiler (http://www.t-profiler.org/), was used. T-Profiler is an online tool for the analysis of gene expression data using the t-test to score the activity of predefined groups of genes [30], [31]. Predictions are based on DNA binding motifs in the promoter region of the corresponding genes. The transcription factors with significant t-values are listed in table 5 and four major groups with common transcriptional regulators were identified.

**Table 5:** T Profiler analysis of TF binding motifs.

TF	As^3+^	Cd^2+^	Hg^2+^	Ag^+^	Ni^2+^	Mn^2+^	Co^2+^	V^3+^	Zn^2+^	Al^3+^	Homeostatic pathway
**Msn2**	9.78	6.46	9.62	3.39	4.47	4.26	2.63	3.56			Stress response
**Msn4**	9.93	6.09	8.2	2.54				3.91			Stress response
**Yap1**	9.39	5.57	7.01	3.18		2.1		2.58			Oxidative stress response
**Yap7**	10.6	5.69	9.02	4.91		3.7					Stress response
**Cad1**	4.46	3.14	4.47	4.87							Stress response
**Yap6**	4.05	2.34	3.22								Stress response
**Hsf1**	14.03	6.21		4.3			2.48	2.66		2.97	Stress response
**Met4**		8.61		3.92				2.86		2.42	Sulfur amino acid biosynthesis
**Met32**		14.49	2.48	7.47		2.49					Sulfur amino acid biosynthesis
**Rpn4**	9.73	5.59	2.22				2.78				Proteasomal degradation
**Gln3**			3.35			11					Nitrogen metabolism
**Gcn4**		2.58	12.68	5.29		17.72					Amino acid biosynthesis
**Rtg3**	2.69	2.61	3.17	4.55		4.95					Mitochondrial degradation
**Put3**					3.82	4.95	8.16	4.32		2.55	Proline utilization
**Aft1**					3.61	4.93	6.88	2.98			Iron homeostasis
**Fhl1**	−16.04	−8.47	−9.6	−13.04	−10.84			−6.15	−5.69		Ribosomal biogenesis
**Sfp1**	−8.5	4.21	−5.32	−6.36	−5.53			−2.65			Ribosomal biogenesis
**Rap1**	−10.09	−5.2	−5.94	−7.75	−5.66			−3.57			Ribosomal biogenesis

Corresponding to TF binding motifs in the upstream region of the detected genes, putative TFs involved in positive and negative response to at least two metal stress conditions were predicted by T-profiler. The numbers are the calculated t-values which are - for any group scored - a measure of the up-regulation (t>0) or down-regulation (t<0) in units of the standard error of the difference.

The largest group comprised the metalloid As^3+^ and the transition metals with no biological role Cd^2+^, Hg^2+^, and Ag^+^ with the key regulators of the yeast stress response Yap1, Msn2, Msn4, and the Yap1 homologues Yap7 and Cad1. This result is consistent with the pair-wise similarity analyses and also with previous studies [15], [32]. In addition, the heat shock factor Hsf1 was predicted as significant for As^3+^, Cd^2+^, and Ag^+^ intoxication and the transcription factor Rpn4, stimulating expression of proteasome genes, for As^3+^ and Cd^2+^ exposure. Met4 and Met32, main factors of the sulfur amino acid regulatory network upon Cd^2+^ and As^3+^ exposure [33], [32], were also predicted to participate in the response to Ag^+^ stress.

A second group included Hg^2+^ and Mn^2+^ which induced genes regulated by the transcriptional nitrogen regulator Gln3 and the amino acid biosynthesis mediating factor Gcn4. Ag^+^ and Mn^2+^ stress activated genes which were enriched by those also targeted by the mitochondrion degradation regulator Rtg3. One further group of Ni^2+^, Mn^2+^, Co^2+^, and V^3+^ induced genes activated by Put3, involved in proline metabolism, and the iron homeostasis factor Aft1.

The transcription factors predicted to be involved in the regulation of the 196 repressed genes under As^3+^, Cd^2+^, Hg^2+^, and Ag^+^, Ni^2+^ and V^3+^ were the regulators of ribosomal biogenesis Fhl1, Sfp1, and Rap1. This result appeared as a significant peak indicative for repression of ribosome biogenesis as the main common early-on transcriptional regulation mechanism to acute metal stress.

**Figure 2:**
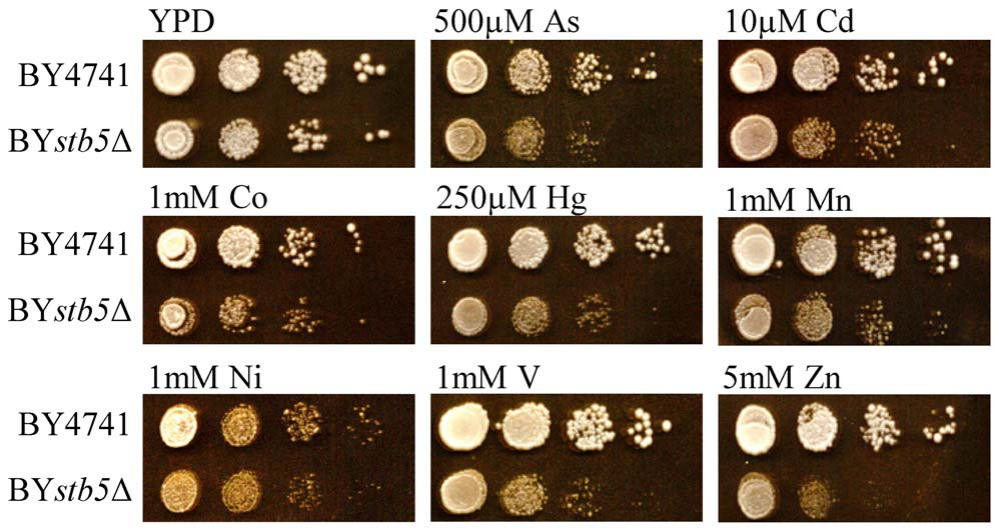
Growth assays with *stb5*Δ mutants. Serial 10-fold dilutions of BY4741 and BY*stb5*Δ cells were spotted onto metal-containing YPD plates (as indicated in the figure) and scored after 48 h.

The mRNA levels of transcription factor genes were mostly unaffected in response to acute metal treatment with *HOT1* and *STB5* mRNAs as the most interesting exceptions. Table 6 summarizes the results for transcription factor genes differentially expressed upon at least two metal stress conditions. Ag^+^, Cd^2+^, and Hg^2+^ induced expression of *HOT1*, encoding a transcription factor required for the transient induction of the glycerol biosynthetic genes *GPD1* and *GPP2*. Hot1 is a direct target of the Hog1 MAP kinase pathway and involved in arsenic resistance [34], [35]. It therefore appears that the Hog1 MAP kinase pathway also obtains a role in the tolerance against other metals than As^3+^. As^3+^ and Cd^2+^ induced transcription of *STB5*, a factor participating in multidrug resistance and oxidative stress response [36], [37]. By means of phenotypic analyses on solid plates supplemented with metal ions it was found that *stb5*Δ mutants exhibited significant growth deficiencies (Figure 2). This indicates an important role of Stb5 in the systemic defense of yeast cells against metal exposure (analysis in preparation).

**Table 6:** Differential expression of TFs under metal stress.

TF	Ag^+^	As^3+^	Cd^2+^	Hg^2+^	Ni^2+^	V^3+^	Mn^2+^	Co^2+^	Zn^2+^	Al^3+^	Homeostatic pathway
**Arg80**					**up**					**up**	Arginine biosynthesis
**Hot1**	**up**		**up**	**up**							Stress response
**Met28**			**up**				**up**				Sulfur amino acid metabolism
**Stb5**		**up**	**up**								Oxidative stress response
**Gat1**								**down**		**up**	Nitrogen metabolism
**Hir1**							**down**		**up**		Histone gene transcription
**Rgt1**			**up**					**down**			Glucose metabolism
**Cha4**						**down**				**down**	Amino acid metabolism

TFs that were found to be significantly up- or down- regulated in at least two metal stress responses.

### Gene ontology analysis for acute metal stress

For identification of generic and specific cellular responses the differentially expressed genes were associated to common GO terms (http://www.yeastgenome.org/cgibin/GO/goTermFinder.pl). The most significant associations are listed in table 7 (additional information is provided in TS 1 and 2 in File S3). Al^3+^ induced genes of the Ty1 retro transposon [38]. Ag^+^ and V^3+^ stress primarily activated the expression of metallothionein (MT) genes (As^3+^ and Zn^2+^ caused a weaker response), whereas Co^2+^ and Zn^2+^ induced especially Aft1-dependent siderophore iron homeostasis genes. As^3+^, Cd^2+^, and Hg^2+^ caused activation of stress response genes encoding proteins involved in protein folding and aldehyde metabolism (As^3+^), in sulfur compound metabolism (Cd^2+^), and in the oxidative stress defense (Cd^2+^ and Hg^2+^). Hg^2+^ and Mn^2+^ led to the induction of Gcn4 and Gln3 regulated genes associated with amino acid metabolism. On the whole this GO annotation matched the associations derived from regulatory analysis (Table 6).

**Table 7:** Significant GO terms associated to the particular metal stress conditions.

Metals	GO terms	Gene names
**Al**	RNA-mediated transposition	*YAR010C,YBL005W-A,YCL020W,YJR026W,YJR028W,YML040W,YML045W*
**Ag**	Metal chelation	*CUP1-1,CUP1-2*
**As**	Protein folding	*SSA1,HSP26,SSA4,SSC1,SSA2,HSP104,CPR6,HSP60,HSC82,YDJ1,STI1,HSP82*
	Aldehyde metabolism	*AAD3,AAD6,AAD16,AAD10,ADH6,AAD14,AAD15*
**Cd**	Sulfur compound metabolism	*CYS3,SAM2, MET2,MET3,MET5,MET6,MET14, MET16,MET17*
	Response to oxidative stress	*YBL055C,PRX1,YDL124W,AHP1,ZWF1*
**Co**	Iron homeostasis	*ARN1,ARN2,FET3,ENB1,FIT2*
**Hg**	Amino acid metabolism	*ARG1,ARG4,CPA2,HIS3,HIS4,LEU1,LEU2,LEU4,FMT1,ARO3,HOM2,HOM3,MET17*
	Response to oxidative stress	*YBL055C,PRX1,GPX2,YDL124W,TRR1,GRX2,TRX2,CCP1,AHP1,TSA1*
	Response to stress	*ATG8,PCL5,CCP1,DDR48*
**Mn**	Amino acid metabolism	*ARG1,ARG4,CPA2,HIS1,HIS3,HIS4,HIS5,HIS7,LEU4,LYS1,LYS9,LYS21,LYS20,*
		*BAT2,ACO1,ORT1,ARO3,TRP2,TRP3,TRP5,TRP4,ILV6,HOM2,MET17,MET22, SAM1,PRO2,GLN1,ASN1,CIT2,IDP1*
	Siderophore iron transport	*ARN1,ARN2,ENB1,FIT2*
**Ni**	No significant GO term	–
**V**	Metal chelation	*CUP1-1,CUP1-2*
**Zn**	Siderophore iron transport	*FIT1,TIS11,FIT2,FIT3*

Genes differentially expressed greater than 2-fold in response to metal stress associated to significant GO terms by GO Term Finder (provided by SGD).

**Figure 3:**
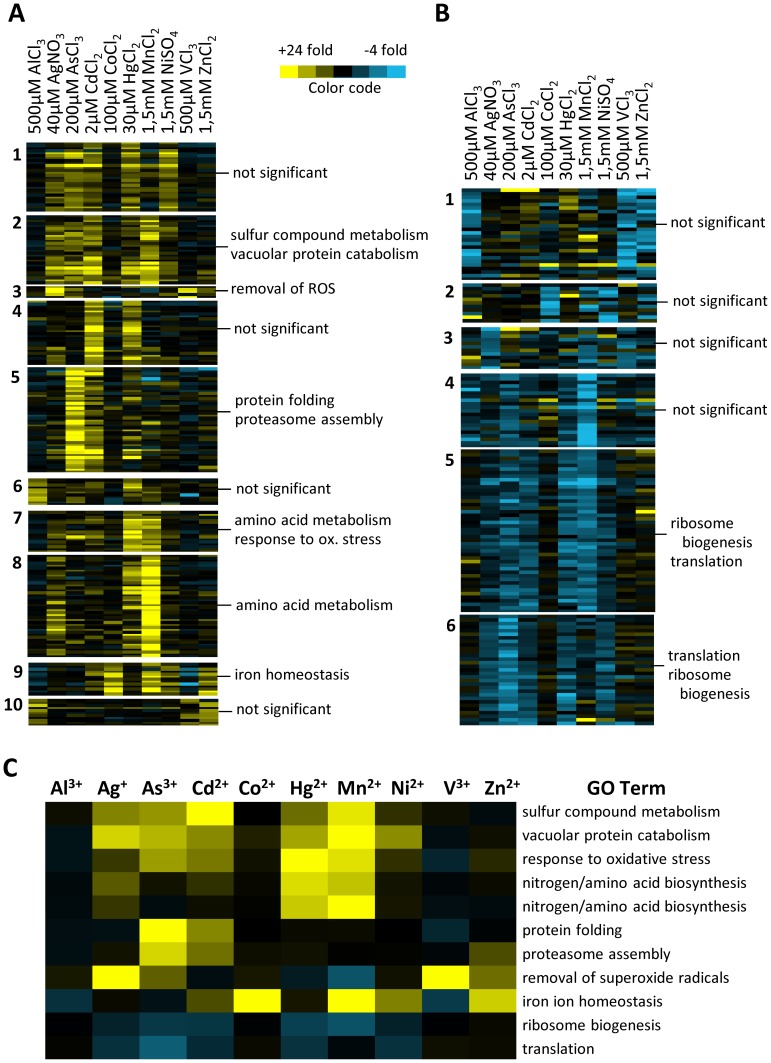
Transcript profile of acute metal stress. Results of the transcriptional metal stress responses following K-means clustering and association to significantly shared GO terms; **A**) with K = 10 for up-regulated genes (yellow); **B**) with K = 6 for down-regulated genes (blue). **C**) Variations in transcript abundance for each significant GO pathway under the particular metal stress conditions were calculated as mean values of fold inductions and repressions. Detailed information is provided in TS 3, 4, 5, 6, and 7 in File S 3.

Genes differentially expressed upon at least two of the 10 metal stress conditions were compared performing K-means clustering, enriched GO terms were amended and the average change of expression for the particular GO term calculated. This allowed us to determine the GO weighted relationships between the different stresses and to generate a graphical representation (Figure 3A and B; additional information is provided in TS 3, 4, and 5 in File S3). Figure 3C illustrates the mean value of fold inductions and repressions of the most significant GO pathways under the particular metal stress conditions (additional information is provided in TS 6 and 7 in File S3). In summary, analyses of transcriptional networks revealed the activation of metal-specific oxidative and general stress defense mechanisms, protein degradation, nitrogen/amino acid biosynthesis, and iron homeostasis along with repression of ribosomal biogenesis and translation as the main response mechanisms of yeast cells upon acute metal stress.

### Common metal ion stress responses

#### Oxidative stress response

**Figure 4:**
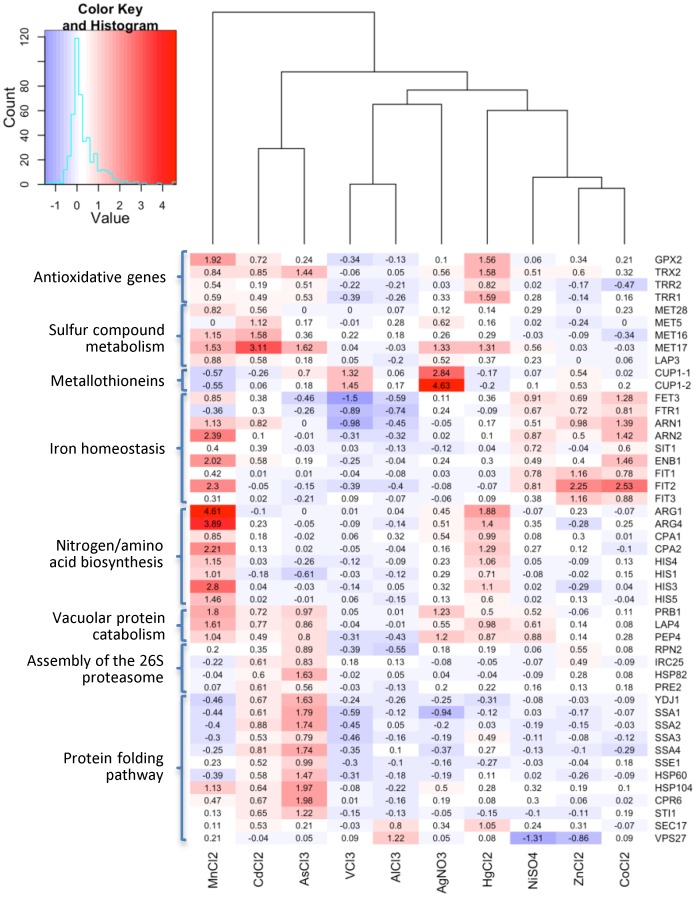
Transcriptional metal defense responses of *S. cerevisiae* to acute metal stress. Two-dimensional hierarchical cluster heat map of the transcriptional profile of genes responding to at least 2 metal stress conditions and being associated to significant GO-Terms; the displayed intensities are the log2 ratios. Differences with expression levels greater than the mean are colored in red and those below the mean are colored in blue. The histogram summarizes the distribution of the fold-changes of all combinations (47 genes and 10 conditions).

The oxidative stress response was a primary candidate for a common metal ion stress response [39], [40], [41], [42]. Indeed, antioxidative genes such as the glutathione peroxidase gene *GRX2* or the cell redox homeostasis genes *TRR1*, *TRR2*, and the before-mentioned *TRX2*, were significantly up-regulated by Hg^2+^ and Mn^2+^ and to a lesser degree by As^3+^ and Cd^2+^ and *TRX2* also by Ag^+^, Ni^2+^, and Zn^2+^. The histogram in figure 4 illustrates these findings. This observation is supportive for the known genotoxic effects of sublethal concentrations of redox-inactive metal ions indirectly mediated by an increase in the reactive oxygen species (ROS) level [43], [44]. One of the main negative characteristics with metals such as As^3+^, Cd^2+^, or Hg^2+^ is their high affinity to thiol (-SH) groups which play a distinct role in the function of several cellular components including enzymes, transcription factors and membrane proteins. These metal ions bind for instance to the ubiquitous antioxidant glutathione and are therefore supposed to indirectly cause oxidative stress by depletion of glutathione [45], [43], [46]. As^3+^, Cd^2+^, Hg^2+^, and Mn^2+^ additionally promoted induction of genes involved in the sulfur compound metabolism (e.g. *MET28*, *MET5*, *MET16*, *MET17*, and *LAP3*; Figure 4). The sulfur/GSH pathway and the thiol redox system (glutathione, thioredoxin) thus appeared to be a general cellular defense against metal stress. This is consistent with recent studies in yeast that reported the conversion of sulfur assimilates into glutathione as a result of As^3+^ and Cd^2+^ exposure [32], [47]. Glutathione acts as a first line of defense against several stresses by sequestering and forming complexes with toxic metalloids [13], [39], [40], [48]. Glutathione conjugates finally can be imported into the vacuole or exported from the cell. The former pathway protects neighboring cells from damage.

Ag^+^ and V^3+^ exposure led to strong, and As^3+^ and Zn^2+^ caused a weaker induction of *CUP1-1* and *CUP1-2* encoding metallothioneins for chelation of toxic metal ions (Figure 4). Both genes have recently been implicated in Ag^+^ stress [49]. This metal detoxification mechanism serves the prevention of metal accumulation and secondarily generated oxidative stress inside the cell [50].

Together, oxidative stress responses were identified for all tested metal ions except for Al^3+^ and Co^2+^. This is probably due to the moderate metal concentrations used in this study because at higher concentrations aluminium and cobalt were shown to induce oxidative stress response as well [51], [52], [53]. Since the applied metal ion concentrations were derived from the growth inhibiting properties, this result may indicate other cellular targets for these metals such as RNA (Al^3+^) or iron homeostasis (Co^2+^). The induction of central elements of the oxidative defense mechanisms by the majority of investigated metal stressors suggested this as the basic detoxification strategy against short-term metal exposure.

#### Iron homeostasis and metal scavengers

Recent studies have shown that metals such as V^3+^, Al^3+^, Co^2+^, Ni^2+^, and Zn^2+^ interfere with iron homeostasis by competing with iron for iron-binding sites of e.g. transporters and other enzymes [54], [55], [56], [57], [58]. In this regard, iron homeostasis genes, especially involved in high-affinity iron transport (*FET3* and *FTR1*) and siderophore iron transport (*ARN1*, *ARN2*, *SIT1/ARN3*, *ENB1/ARN4*, *FIT1*, *FIT2*, and *FIT3*; Figure 4) were induced in response to Co^2+^, Mn^2+^, Ni,^2+^ and Zn^2+^. Notably, a recent study reported that siderophores can reduce metal toxicity [59]. Induction of the siderophore ion transport system might therefore serve as one further general detoxification mechanism by chelating extracellular metal ions to prevent their uptake.

Metal scavengers provide an additional defense mechanism. Significant up-regulation of nitrogen/amino acid biosynthesis genes mainly including arginine (*ARG1*, *ARG4*, *CPA1*, and *CPA2*) and histidine (*HIS1*, *HIS3*, *HIS4*, and *HIS5*) was observed in response to Mn^2+^ and Hg^2+^ (Figure 4). For *S. cerevisiae* it has been shown that the accumulation of histidine in the vacuole decreases the toxicity of copper, cobalt and nickel [60], [61]. It has been proposed that histidine prevents zinc toxicity by chelating zinc in mammalian cells [62]. Accordingly, we suggest chelation of Mn^2+^ and Hg^2+^ via histidine as an important detoxification strategy in yeast as well. Whether this might also be true for arginine is yet unclear.

#### Protein catabolism

Degradation of damaged proteins appeared to significantly contribute against acute metal stress. The transcript profiles revealed strong induction of vacuolar protein catabolism genes (*PRB1*, *LAP4*, and *PEP4*; Figure 4) in response to Ag^+^, As^3+^, Cd^2+^, and Mn^2+^ and to a lesser degree to Hg^2+^, and Ni^2+^. Genes (*RPN2*, *IRC25*, *HSP82*, and *PRE2*; Figure 4) involved in the assembly of the 26S proteasome, responsible for non-vacuolar degradation of cellular proteins [63] were significantly activated under As^3+^ and Cd^2+^ exposure. This treatment caused also a considerable induction of the protein folding pathway (*YDJ1*, *SSA1*, *SSA2*, *SSA3*, *SSA4*, *SSE1*, *HSP60*, *HSP104*, *CPR6*, and *STI1*; Figure 4) with the formation and activation of chaperone complexes for folding and refolding of proteins [64], [65], suppression and rescue of protein aggregates [66], [67], and degradation of aberrant proteins [68].

*SEC17*, encoding a membrane protein required for vesicular transport and autophagy was induced in response to Hg^2+^ and to a lesser degree to Al^3+^ and Cd^2+^. Yeast cells employ the catabolic process of autophagy to degrade damaged or obsolete organelles and proteins. This is consistent with a study reporting that the yeast Sec19 vesicle transport system accounts for increased metal tolerance [69]. Al^3+^ also up-regulated *VPS27* involved in ubiquitin-dependent protein catabolism in the vacuole.

#### Repression of protein synthesis

The majority of repressed genes upon Ag^+^, As^3+^, Cd^2+^, Hg^2+^, Mn^2+^, and Ni^2+^ exposure were involved in ribosomal biogenesis and translation (Figure 3). This has been noted also in previous studies [15], [70], [71]. In rapidly growing yeast cells ribosomal protein mRNAs contribute to nearly 30% of the total mRNAs and therefore constitute major consumers of cellular resources [72], [73]. Therefore, the restriction of ribosomal protein gene expression is suggested to divert the cellular resources in favor of activation of metal defense. Alternatively, high demand of Pol II for stress gene transcription might transiently limit resources for ribosomal gene expression.

Ribosomal protein gene transcription is regulated by the evolutionarily conserved target of rapamycin (TOR) pathway, which mediates growth control in all eukaryotes [74], [75]. Previous reports further implicated Fhl1 and Rap1 in the regulation of ribosomal biogenesis [32], [76], [77], [78]. In addition, it has recently been shown that As^3+^, Hg^2+^, and Ni^2+^ stress inactivates Sfp1, a transcription factor for ribosomal protein genes, and leads to subsequent reduction of gene transcription [71]. Indeed, T Profiler analysis predicted these TFs, Sfp1, Fhl1 and Rap1, as negative regulators for most metal ion stresses used (except Mn^2+^; Table 6). Direct effects of all three TFs on the regulation of ribosomal protein gene transcription during metal ion stress, however, remain to be determined.

### Metal stress profile changes during adaptation (up to 2 h)

To prove the uniqueness of our results we compared our profiling data (40 µM AgNO_3_, 200 µM AsCl_3_, 2 µM CdCl_2_, 30 µM HgCl_2_, 1,5 mM ZnCl_2_) of acute metal stress (30 min) to those of sustained metal stress (2 h) obtained previously (20 µM AgNO_3_, 400 µM NaAsO_2_, 5 µM CdCl_2_, 19 µM HgCl_2_, and 1 mM ZnSO_2_; [16]; additional information is provided in TS 1 and 2 in File S4). Although different metal concentrations and, in some cases, also different metal salts (arsenic and zinc) had been used in both studies we examined as a matter of principle whether there might be striking differences between these expression profiles. In previous studies we found exactly the same transcriptional changes in response to NiCl_2_ and NiSO_4_ (data not shown) which might also be true for ZnCl_2_ and ZnSO_4_. Whether this also applies to AsCl_3_ and NaAsO_2_ is unclear.

**Figure 5:**
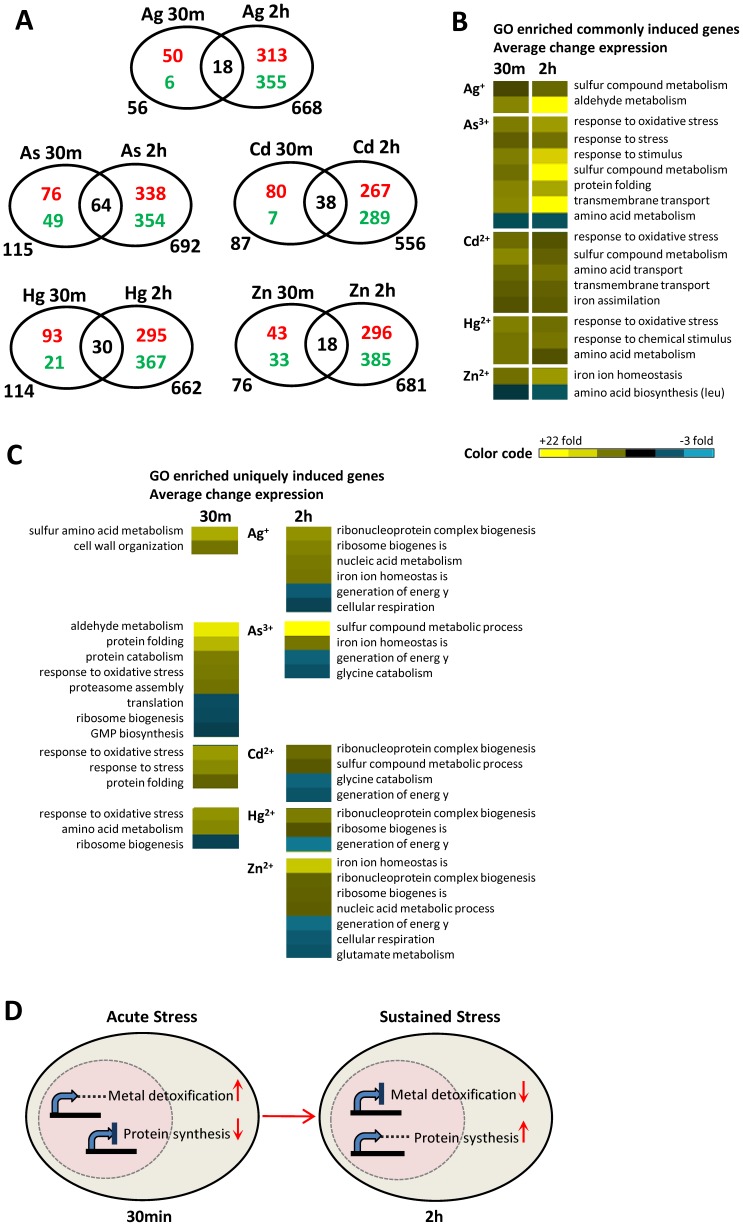
Comparison of expression patterns upon acute and sustained metal stress. **A**) Venn diagrams illustrate the distribution of transcriptionally up-regulated (red) and down-regulated (green) genes from metal-stressed BY*wt* cells (30 min - acute stress; 2 h – sustained stress; additional information is provided in TS 1 and 2 in File S4). **B**) and **C**) Analysis of genes of both data sets via T Profiler associated to significant GO terms; variations in transcript abundance for each significant GO pathway under the particular metal stress conditions were calculated as mean values of fold inductions and repressions; up-regulated genes (yellow), down-regulated genes (blue); detailed information is provided in TS 3, 4, 5, and 6 in File S4. B) overlapping genes; C) genes expressed in one set. **D**) In response to acute metal stress (30 min) transcription of protein synthesis is inhibited (↓) to divert energy to the transcription of metal detoxification (↑). Under sustained metal stress (2 h) transcription of metal detoxification pathways is deactivated (↓), whereas protein synthesis is reactivated (↑).

Interestingly, Venn analysis showed a merely small overlap of 37% of genes changed (greater/minor than 1.2-fold) under acute metal stress with 5% from sustained metal stress. The smallest overlap was found with Ag^+^ and Zn^2+^ (18 genes), followed by Hg^2+^ (30), Cd^2+^ (38), and As^3+^ (64) (Figure 5A). Irrespective of the slightly different metal concentrations used in both studies this result was indicative for constitutive transcriptional changes between acute and sustained metal stress.

T Profiler analyses showed that the overlapping genes of both data sets coded for proteins involved in the oxidative and general stress response, in sulfur and amino acid metabolism, in transport processes, and in iron homeostasis (Figure 5B; additional information is provided in TS 3 and 4 in File S4). These metabolic pathways appear to comprise the essential stress response of yeast cells under acute and sustained metal exposure.

While acute metal ion stress (30 min) induced genes specific for cell wall organization, amino acid biosynthesis, chaperones as well as proteolysis and repressed genes involved in translation and ribosome biogenesis, sustained metal treatment evoked the up-regulation of additional iron homeostasis and sulfur compound metabolism genes, and genes involved in nucleic acid metabolism along with the down-regulation of energy generation and respiration genes. We also observed detoxification pathways in response to acute metal stress, which were not present after 2 h (Figure 5C; additional information is provided in TS 3, 5, and 6 in File S4). Growth-related processes such as protein synthesis that were initially repressed were reactivated upon continued metal treatment (except under 1250 µM As^3+^; [16]). Remarkably, sustained metal exposure showed a reciprocal correlation to the transcriptional activity of metal detoxification and protein synthesis under acute metal stress as illustrated in Figure 5D. Taken together, these results indicate transcriptional adaptation of yeast cells under prolonged metal treatment. However, whether yeast cells indeed adapt to sustained metal stress still needs to be evidenced by long-term experimental setup (in preparation).

## Conclusions

We addressed the question of whether stress evoked by different metal ions will cause an early-on generic cellular response pattern or if specialized metal dependent mechanisms prevail. Therefore the immediate early stress response of *S. cerevisiae* cells to ten biologically relevant metals with Cd^2+^ as the most toxic one, followed by Hg^2+^, Ag^+^, As^3+^, Co^2+^, Al^3+^, V^3+^, Ni^2+^, Mn^2+^, and Zn^2+^ was investigated. About 15% of the yeast transcripts were found differentially expressed within 30 minutes. Analyses of transcript profiles and the respective regulatory associations revealed some common processes for distinct groups of metals with shared transcriptional regulators while also unique expression patterns for particular metals were evident. However, yeast cells appear to exhibit no generic detoxification pathway against metal ions.

**Figure 6:**
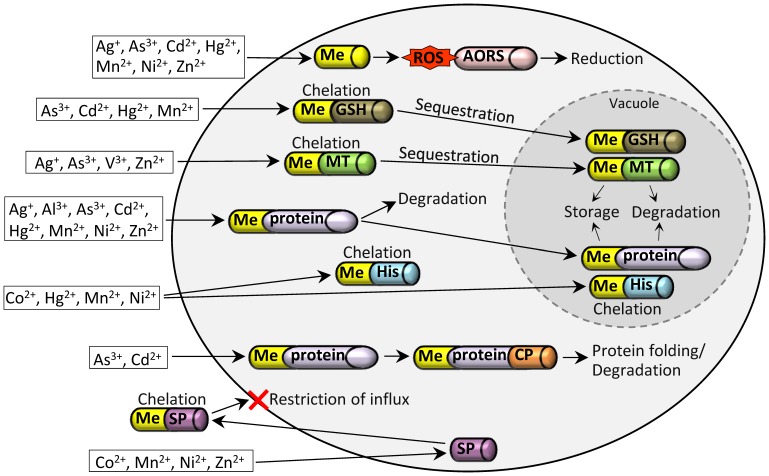
Schematic model of detoxification responses under acute metal stress. Activation of the antioxidative redox system (AORS) to reduce reactive oxygen species (ROS); Chelation of metal ions (Me) via glutathione (GSH) and metallothionein (MT), sequestration of chelates into the vacuole, storage of metal ions and degradation of proteins, respectively; Extracellular chelation of metals via siderophores (SP) to restrict metal influx; Chelation of metals via histidine (His); Vacuolar and non-vacuolar degradation of metal/protein complexes; Activation of chaperones (CP) for protein folding and degradation of metal/protein complexes.

The primary transcriptional response to all tested metal ions comprised activation of metal-specific oxidative defense and protein degradation processes, most likely to remove damaged cellular components and to prevent secondary damage. The detected antioxidant responses mainly included the glutathione/thioredoxin and metallothionein system, whereas catabolic processes during acute metal stress involved vacuolar protein degradation, proteasomal proteolysis, chaperone complex activities as well as Sec19 vesicle transport. Additional putative metal-specific detoxification strategies such as chelation of metal ions via siderophores and histidine were observed. Figure 6 summarizes all metal detoxification strategies in response to acute metal stress.

Reduction of genes coding for ribosomal biogenesis and translation was found as the main common gene repression effect under acute metal exposure. Comparison of our set with a compendium profile after 2 h metal exposure showed reactivation of protein synthesis and down-regulation of detoxification pathways indicating rapid adaptation processes to acute metal exposure via effective establishment of metal defense on a proteomic level. Chronic metal exposure has been reported recently to result in the formation of new epigenotypes providing increased resistance to metal ions [79].

In summary, the results of our metal stress study demonstrated specific detoxification mechanisms to distinct metal stress conditions, deciphered dedicated regulatory coherences under metal stress, and itemized metabolic changes during adaptation to metal stress. These investigations may thus support the available data pool for characterization of the general stress response which has received considerable attention in view of further usage of model organism in toxicity testing implications. However, a number of questions still remain open. Of particular importance will be a systematic study of the highly sophisticated cross-regulations of innumerable transcriptional as well as posttranslational regulators involved in metal toxicity and detoxification processes.

## Supporting Information

File S1 **Table S1**. Metals selected for this study. Biological role, biotechnical uses, and health risks of the metals (M); Selected metals: silver (Ag), cadmium (Cd), cobalt (Co), mercury (Hg), manganese (Mn), nickel (Ni), vanadium (V), zinc (Zn), arsenic (As), and aluminium (Al); Used internet sources: Rutherford – Lexikon der Elemente (http://www.uniterra.de/); Web Elements – The Periodic Table (http://www.webelements.com/); Wikipedia, the free encyclopedia (http://www.wikipedia.org/); ATSDR – Agency for Toxic Substances and Disease Registry (http://www.atsdr.cdc.gov/).
(DOC)

File S2 **TS 1 Figure 1**. Unselected data sheet (all genes). **TS 2 Figure 1**. Significantly up-regulated genes (>1.5-fold). **TS 3 Figure 1**. Significantly down-regulated genes (<0.5-fold). **TS 4 Table 2**. Up-regulated genes. Analysis of gene expression (greater than 50% induction) overlaps between the transcriptional profiles. **TS 5 Table 2**. Down-regulated genes. Analysis of gene repression (minor than 50% repression) overlaps between the transcriptional profiles. **TS 6 Table 3**. Genes uniquely ex-/repressed upon the distinct metal stress conditions. **TS 7 Table 4**. Analysis of all possible gene ex-/repression overlaps between the transcriptional profiles.
(XLS)

File S3 **TS 1 Table 7**. Genes 2-fold up- or down-regulated. **TS 2 Table 7**. Genes (differentially ex/repressed greater/minor than 2-fold) associated to significant GO terms by GO-Term Finder: GO-Term Finder searches for significantly shared GO terms of the clustered genes; **TS 3 Figure 3A**. K-means clustering of the transcription profiles was performed with K = 10 for up-regulated genes. **TS 4 Figure 3B**. K-means clustering of the transcription profiles was performed with K = 6 for down-regulated genes. **TS 5 Figure 3A and B**. GO Term Finder up- and down-regulated genes. **TS 6 Figure 3C**. Genes up-regulated (greater than ∼1.5-fold) upon metal stress. **TS 7 Figure 3C**. Genes down-regulated (minor than ∼1.5-fold) upon metal stress.
(XLS)

File S4 **TS 1 Figure 5**. Profiling data Hosiner - genes expressed greater/minor than 1.5-fold. **TS 2 Figure 5**. Profiling data Jin - genes expressed greater/minor than 1.5-fold. **TS 3 Figure 5B and C**. T Profiler analysis; Jin genes in comparison with Hosiner genes (all genes expressed greater/minor than 1.5-fold) associated to significant GO terms. **TS 4 Figure 5B**. Hosiner/Jin gene overlaps (expression greater/minor than 1.2-fold in both experimental setups) associated to significant GO terms by T Profiler. **TS 5 Figure 5C**. Genes which were solely expressed (greater/minor than 1.5-fold) in the Hosiner data set associated to significant GO terms by T Profiler. **TS 6 Figure 5C**. Genes which were solely expressed (greater/minor than 1.5-fold) in the Jin data set associated to significant GO terms by T Profiler.
(XLS)
