# Supplementary material for: Impact of Acute Metal Stress in Saccharomyces cerevisiae
Source: PLoS One. 2014 Jan 9;9(1):e83330. doi: 10.1371/journal.pone.0083330 (PMC3886979; doi:10.1371/journal.pone.0083330)
Supplement: File S1 — Table S1. Metals selected for this study. Biological role, biotechnical uses, and health risks of the metals (M); Selected metals: silver (Ag), cadmium (Cd), cobalt (Co), mercury (Hg), manganese (Mn), nickel (Ni), vanadium (V), zinc (Zn), arsenic (As), and aluminium (Al); Used internet sources: Rutherford – Lexikon der Elemente (http://www.uniterra.de/); Web Elements – The Periodic Table (http://www.webelements.com/); Wikipedia, the free encyclopedia (http://www.wikipedia.org/); ATSDR – Agency for Toxic Substances and Disease Registry (http://www.atsdr.cdc.gov/). (DOC) [file pone.0083330.s001.doc]

| **M** | **Biological role** | **Biotechnological uses and**  **Sources of exposure** | **Health risks** |
| --- | --- | --- | --- |
| **Ag** | no biological role; | jewelry, photography, electrical contacts, batteries, silverware, used in mirror production, coinage metal,dental amalgam, antibacterial agent; | most Ag-salts are toxic (esp. to lower organisms); skin irritation; renal and reproductive disorders; hemolysis; nervous system impairment; |
| **Al** | no certain biological role;  may be involved in the action of some enzymes (e.g. succinic dehydrogenase); | cans and foils, kitchen utensils, alloys, industrial applications, electrical transmissions, antacids, buffered aspirin, nasal sprays, antiperspirants, automobile exhaust, tobacco smoke; | toxic; may increase the risk of breast cancer; deposition in bone and the central nervous system; linked to Alzheimer´s disease; nervous and digestive disorders; musculoskeletal and respiratory problems; |
| **As** | no biological role in higher organisms; plays an essential role in some species of bacteria; | paints, pyrotechnics, glasses, wood preservatives, alloys, rat poisoning, fungicides, pesticides, coal combustion, table salt, drinking water, beer,; | potent poison and carcinogenic; renal, gastrointestinal and respiratory problems; neurological, hepatic and reproductive disorders; |
| **Cd** | no biological role in higher organisms; plays a necessary role in marine diatoms; | instant dental alloys, paints, ceramics, batteries, PVC plastics, fungicides, insecticides, marijuana, rubber, seafood (oyster, tuna), coffee, mushrooms, cigarettes, liver, exhaust; | extremely toxic and carcinogenic (e.g. lung and prostate cancer); renal failure and blood pressure problems; neurological disorders; gastrointestinal symptoms; respiratory and reproductive problems; |
| **Co** | essential trace element (coenzymes – cobalamins; e.g. vitamin B_12_); deficiencies of cobalt lead to pernicious anemia; | radio therapeutic, alloys, magnet steels and stainless steels, electroplating; ceramics, glass, pottery, batteries, paints; | most components are toxic, some are considered carcinogenic; contact dermatitis; cardiovascular and respiratory difficulties; developmental problems; overproduction of red blood cells; |
| **Hg** | no biological role; | antifouling paint, thermometers, batteries, dental amalgams, contact lens solutions, pesticides, childhood vaccines, antiseptic creams, sea fish; | extremely toxic; nervous and digestive disorders; developmental disorders; allergies; infertility; ocular and renal problems; |
| **Mn** | essential trace element (enzymes); deficiencies lead to infertility, metabolic weakness, pancreas and enzyme problems; | oxidizing agent, medicine, nuts, seeds, whole-grain products, beans, peas, ginger, coffee, tea, wheat germ; | highly toxic; possibly carcinogenic (e.g. fibroid tumors); nervous and cardiovascular disorders; respiratory problems; osteoporosis; liver diseases; diabetes; |
| **Ni** | essential trace element for many species (bacteria, plants);  deficiencies in rats and chicks lead to liver problems; | , alloys, coinage metal, glasses (green color), catalyst, batteries, jewelry, electroplating, cigarette smoking,, peanut butter, hydrogenated vegetable oils, tea, margarine, herring, oysters; | toxic (esp. for plants); carcinogenic (esp. lung);  skin allergies (dermatitis); digestive and respiratory disorders; cardiac symptoms (Ni interferes with vitamin E activity); immunological problems; kidney damages; |
| **V** | essential trace element for some species (e.g. rats, chicks);  deficiencies result in reduced growth and impaired reproduction; | plastics, nuclear applications, bonding agent, ceramics, industrial smoke pollution, diesel fuels, vegetable oils, olives, black pepper, seafood, spinach; | highly toxic; possibly carcinogenic (esp. lung);  arthritis, cardiovascular and renal problems; weakened immune system; gastrointestinal and respiratory problems; reproductive disorders; |
| **Zn** | essential trace element (enzymes); deficiencies result in skin, hair and nail problems, depression, stunted growth and male infertility, immune deficiency, hypothyroidism, | paints, rubber and dyes, dry batteries, lightweight coins, plastics, textiles and electrical equipments, fluorescent lights; industrial smoke, cosmetics, pharmaceuticals; | skin irritant; affects the balance of other nutrients (e.g. Ni, Ca, vitamin A, B1, C); gastrointestinal and respiratory problems; hematological disorders; interstitial nephritis; |
